# General Validity of Levelt's Propositions Reveals Common Computational Mechanisms for Visual Rivalry

**DOI:** 10.1371/journal.pone.0003473

**Published:** 2008-10-22

**Authors:** P. Christiaan Klink, Raymond van Ee, Richard J. A. van Wezel

**Affiliations:** 1 Functional Neurobiology & Helmholtz Institute, Utrecht University, Utrecht, the Netherlands; 2 Department of Physics and Helmholtz Institute, Utrecht University, Utrecht, the Netherlands; Istituto di Neurofisiologia, Italy

## Abstract

The mechanisms underlying conscious visual perception are often studied with either binocular rivalry or perceptual rivalry stimuli. Despite existing research into both types of rivalry, it remains unclear to what extent their underlying mechanisms involve common computational rules. Computational models of binocular rivalry mechanisms are generally tested against Levelt's four propositions, describing the psychophysical relation between stimulus strength and alternation dynamics in binocular rivalry. Here we use a bistable rotating structure-from-motion sphere, a generally studied form of perceptual rivalry, to demonstrate that Levelt's propositions also apply to the alternation dynamics of perceptual rivalry. Importantly, these findings suggest that bistability in structure-from-motion results from active cross-inhibition between neural populations with computational principles similar to those present in binocular rivalry. Thus, although the neural input to the computational mechanism of rivalry may stem from different cortical neurons and different cognitive levels the computational principles just prior to the production of visual awareness appear to be common to the two types of rivalry.

## Introduction

In a world that provides an abundance of visual information our brain seemingly effortlessly decides which information reaches awareness. In the lab, this process can be studied using stimuli that cause perception to alternate between competing interpretations while staying constant on the retina [1]. Two categories of such stimuli can be distinguished. In binocular rivalry the two eyes are independently presented with different visual stimuli (e.g. dissimmilarly oriented gratings), causing either eye's image to be perceived in turn. In perceptual rivalry visual information is the same for both eyes but rivalry arises due to the existence of multiple mutually exclusive perceptual interpretations of the stimulus. An example is the well-known Necker cube, which causes perception to alternate between two spatial organizations of a flat line drawing. Binocular and perceptual rivalry are both manifestations of how the visual system handles inconclusive sensory evidence, but it remains unclear whether they include common computational mechanisms.

A comparison of the two types of rivalry tells us that their phenomenological appearance [2] and temporal dynamics [3], [4] are similar during continuous viewing. Both types of rivalry can, in a qualitatively similar -yet quantitatively different- manner, be influenced by attentional efforts to hold one of the two alternative percepts dominant [5], [6]. Furthermore, when presented with intermittent blank periods, they exhibit qualitatively identical effects of stimulus timing [7], [8], [9], and voluntary control [9]. Together, this suggests that even though binocular and perceptual rivalry may arise at different cortical levels (causing quantitative differences), the computational rules to produce perceptual output may be common (causing qualitative similarities).

Several computational models are available that provide an explicit theory of the computational mechanisms that underly binocular rivalry [8], [10], [11], [12], [13]. An important set of constraints for binocular rivalry models are based on the observations by Levelt [14] regarding the relation between the strength (contrast) of the eyes' images and the time course of perceptual alternations. Levelt described in four propositions how perceptual dominance durations are affected by changes in the contrasts in either or both of the images engaged in rivalry. For instance, if the contrast of one image is increased this provides a competitive advantage to the associated neural representation, leading to a greater predominance of the corresponding percept (Levelt's rule I). Levelt's complete set of binocular rivalry propositions state that 1) *Increasing the stimulus strength in one eye will increase the predominance of the stimulus*; 2) *Increasing the stimulus strength in one eye will not affect the average duration of dominance in that eye*; 3) *Increasing the stimulus strength in one eye will increase the rivalry alternation rate*; 4) *Increasing the stimulus strength in both eyes will increase the rivalry alternation rate* [14]. More recent observations, dictate a critical re-evaluation of the second proposition [15], [16], [17]. Levelt's second proposition appears to be valid for high-contrast binocular rivalry stimuli, but to reverse for low-contrast stimuli [17]. This means that based on this existing literature the second rule can no longer be regarded as valid and should be rephrased as *‘manipulations of stimulus strength mainly influence the dominance durations of the percept from the eye with the strongest stimulus’*. We will refer to this new rule as ‘the revised second proposition’.

It is currently unclear to what extent theories that have been developed for binocular rivalry can be applied to other forms of rivalry. In this study we investigate whether Levelt's psychophysical observations that lie at the basis of virtually all binocular rivalry models can be generalized to perceptual rivalry. We use a structure-from-motion stimulus (for a review see [18]) for which a two-dimensional projection of a transparent sphere revolving around a vertical axis gives rise to perceptual rivalry between two depth organizations. In the absence of explicit depth information the sphere is perceived to alternately rotate in either of two directions: with the leftward moving surface in front and the rightward moving surface in the back, or vice versa. We investigate how the time course of perceptual alternations between these rotation directions is affected by changes in the luminance of the dots that define either of the two surfaces. Analogous to binocular rivalry, where an increase in the contrast of one of the conflicting images alters the neural competition process in favor of the corresponding neural representation, a luminance increment of the dots that comprise one of the surfaces in structure-from-motion rivalry alters the competition process such that the brighter surface is perceived in front a larger fraction of the time [19], [20]. However, it is an open question whether these manipulations – image contrast in binocular rivalry and dot luminance in structure-from-motion rivalry – affect the competition process in similar ways, or whether they are different.

Our results demonstrate that all four propositions regarding contrast and perceptual dynamics in binocular rivalry can without any serious modification be applied to dot luminance in bistable structure-from-motion. An important implication of this finding is that models of binocular rivalry that were inspired by Levelt's propositions can be applied to structure-from-motion rivalry as well. Moreover, given the highly dissimilar nature of the ambiguity in these two forms of rivalry, our results suggest that the neural computations that produce dominance in visual rivalry share common features for a broad range of rivalry stimuli.

## Materials and Methods

### Observers

Five observers with normal or corrected to normal vision, ranging in age between 21 and 28 years, participated in our experiment. One of the observers was an author (CK), but the other four (students) were completely naïve with respect to the aims of the study.

### Apparatus

Visual stimuli were generated on a Macintosh computer in MATLAB (MathWorks, Natick, MA) using the Psychtoolbox extensions [21], [22] and presented on a 22 inch CRT monitor with a resolution of 1280×1024 pixels and a refresh rate of 100 Hz. Observers used a head- and chinrest and viewed the stimuli from a distance of 120 cm.

### Stimulus and procedure

**Figure 1:**
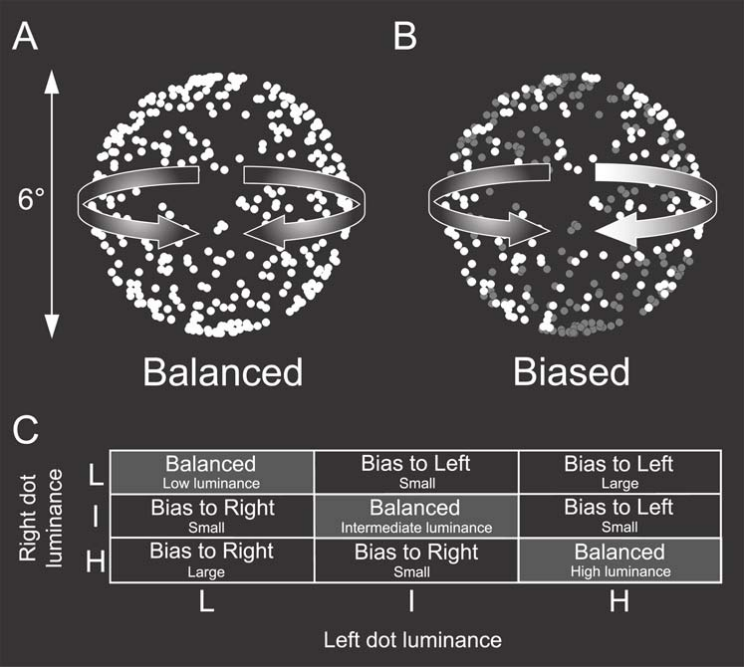
Schematic representation of the stimulus. White dots on a black background moving with a sinusoidal speed profile create the impression of a three-dimensional sphere rotating in depth around the vertical axis. If all dots have the same luminance (A, Balanced) both rotation directions are equally likely and the sphere is perceived to switch rotation direction every few seconds. If the dots moving in opposite directions have a different luminance (B) the sphere is biased towards the perceptual interpretation with the brightest dots in the foreground. (C) In our experiment we used three different luminance levels (Low = L, Intermediate = I, High = H) for the two surfaces resulting in nine different sphere stimuli.

The stimulus was a bistable rotating sphere, composed of two transparent layers of 450 random white dots with a sinusoidal speed profile on a black background (0.0 cd/m^2^). The sphere size was 6 degrees, the dot size 0.05 degrees and the rotation speed was 80 degrees per second. The number of dots and rotation velocity were chosen to maximize the number of reversals [23]. The luminance of the dots of both surfaces was manipulated between low, intermediate and high white intensities (corresponding to 25.3, 41.3 and 61.2 cd/m^2^ respectively). This resulted in nine (3×3 dot luminance values) sphere stimuli configurations (figure 1). The dots at the two different luminance values were drawn on the screen in random order to avoid a true depth ordering (due to overlapping dots) of the two layers. Stimuli were pseudo randomly chosen form the nine possible configurations and presented for 300 seconds while observers reported the perceived rotation direction of the sphere by pressing one of two buttons on a keyboard. Observers were explicitly instructed to report the direction of the perceived front surface to minimize the role of mixed percepts [24]. Transition periods between two percepts were not recorded but subjects indicated that they were very short if present at all.

### Data analysis

From the reported perceptual episodes we calculated the average dominance duration, reversal rates and predominance (percentage time spent in one percept) for all experimental conditions. As an extra test, percept durations were fit to a cumulative gamma-rate distribution function [4] using a bootstrap routine (1,000 repeats) to more reliably estimate the mean dominance durations. A Kolmogorov-Smirnov analysis demonstrated that more than 92 percent of our fits were significant at p = 0.05. Because all statistical analyses on the data yielded similar results for the directly calculated and fitted mean dominance durations we only report the results for the directly calculated percept durations. Group data were normalized to an observer's mean percept duration during the intermediate balanced luminance condition or mean reversal rate over all conditions. Statistical differences between conditions were tested with one-way analyses of variance (ANOVA).

## Results

To systematically evaluate the validity of the four propositions for perceptual rivalry we need to make a small -merely semantic- change to Levelt's original propositions. Stimulus strength and perceptual interpretation are tightly coupled in perceptual rivalry, but unlike in binocular rivalry they are not exactly similar. We updated the propositions accordingly and the results of our experiments will be presented following the original order of the propositions. Importantly, in the balanced stimulus conditions none of our observers demonstrated a significant bias for either of the two rotation directions of the bistable sphere (ANOVA, p>0.27).

### • Proposition 1: Increasing the stimulus strength of one perceptual interpretation of a bistable stimulus increases the predominance of this perceptual interpretation

**Figure 2:**
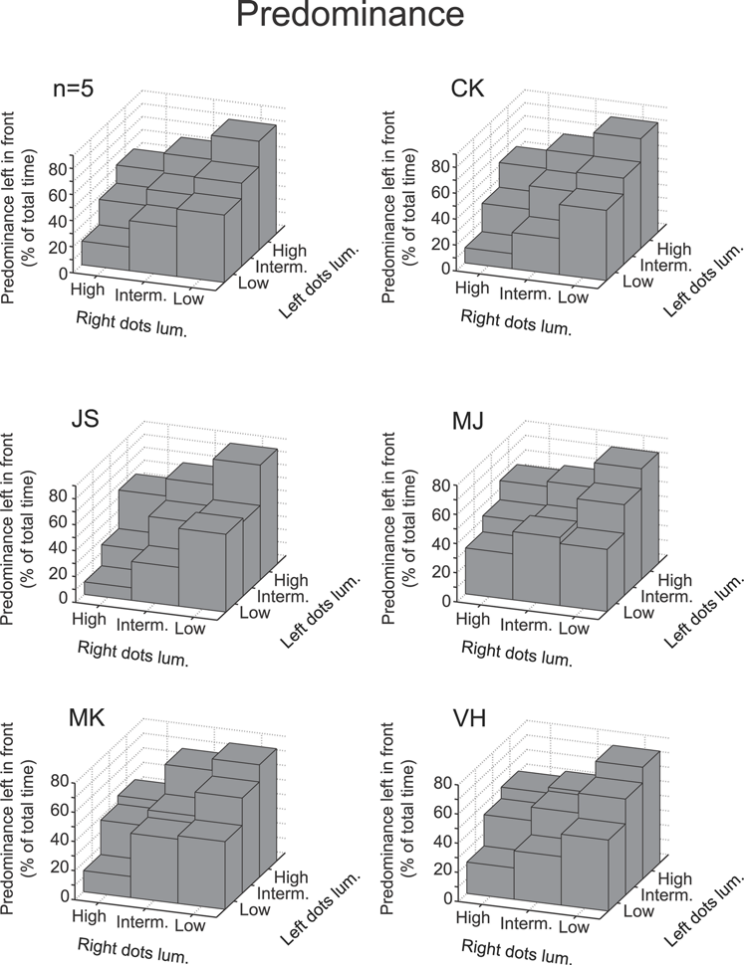
Predominance as a function of individual surface dot luminance. For both the group data (n = 5) and individual observers a balanced increase of stimulus luminance (the diagonal) does not affect the predominance. If the luminance of only one layer of dots is manipulated the predominance shifts towards the perceptual interpretation with the brightest dots in the foreground.

Observers are more likely to perceive the surface with the brighter dots in the foreground and this effect is more prominent for larger dot luminance differences. Statistical analysis of the predominance data revealed significant increases of predominance (percentage of the total time that a percept is dominant) with increasing dot luminance for both left and rightwards moving dots (Left: F(2,12) = 36.56, p<0.001; Right: F(2,12) = 20.28, p<0.001). Likewise, decreasing dot luminance led to significant decreases in predominance of the corresponding percept (Left: F(2,12) = 31.73, p<0.0001; Right: F(2,12) = 28.35, p<0.0001). Balanced dot luminance manipulations did not have any significant effect on predominance (F(2,12) = 0.19, p = 0.83). Figure 2 shows the predominance of leftward rotating spheres (left in front) for all combinations of dot luminance. A similar pattern is present for all observers and the average group data (top left panel). These findings confirm that the perception of a bistable sphere is consistent with the first proposition.

### • (Revised) proposition 2: “Manipulations of stimulus strength of one perceptual interpretation of a bistable stimulus will mainly influence the average dominance duration of the perceptual interpretation corresponding to the strongest stimulus”

Increased predominance of a percept can be the result of longer dominance durations of the percept, shorter dominance durations of the opposite percept or both. Recently it has been shown that changes in predominance in binocular rivalry mainly results from changes in the average dominance duration of the strongest stimulus [17]. Our bistable rotating sphere demonstrates similar results for perceptual rivalry confirming our revised second proposition for perceptual rivalry.

**Figure 3:**
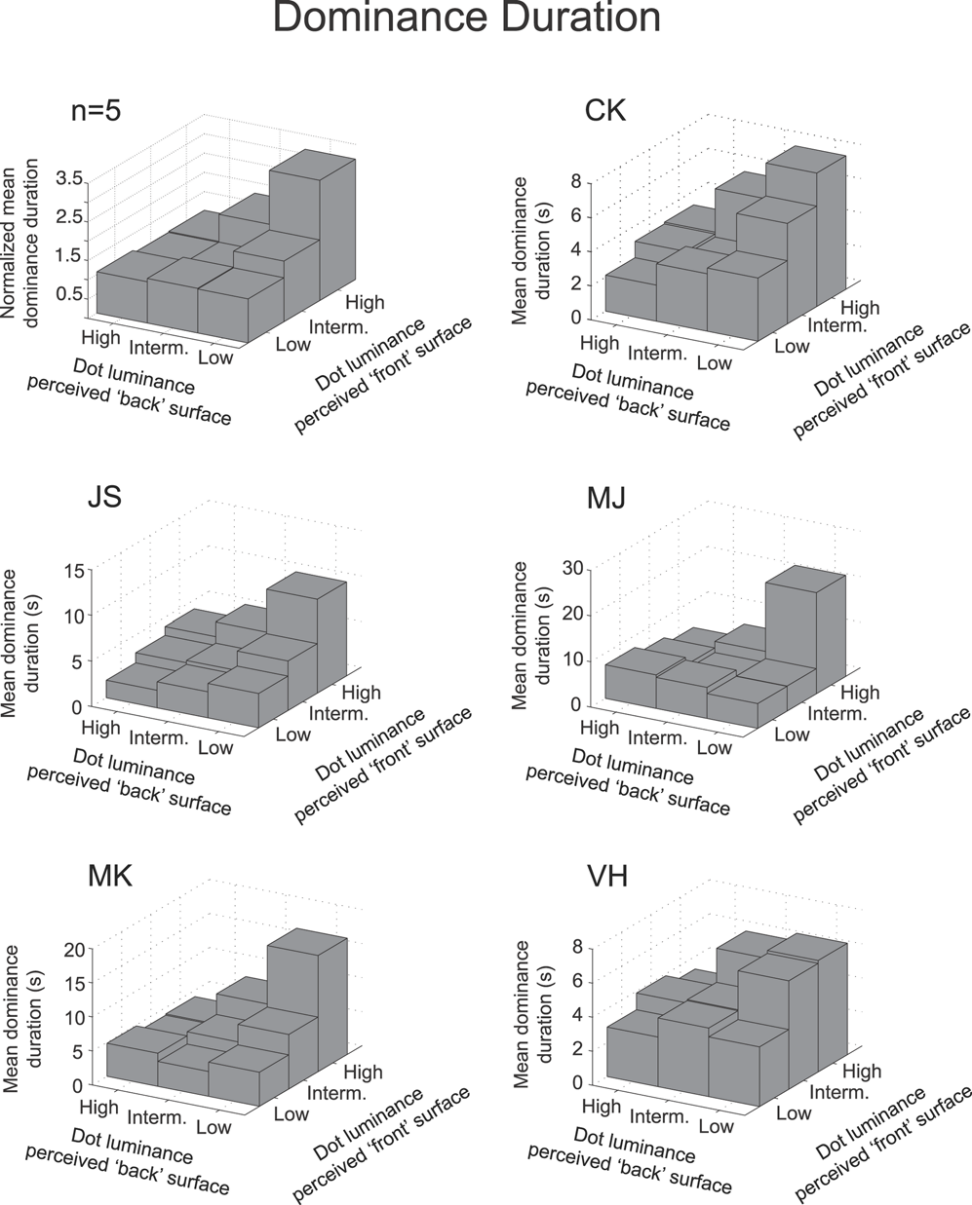
Percept dominance durations as a function of differential dot luminance. Dominance durations are plotted for percepts with defined luminance for the perceived ‘front’ and ‘back’ surface of the sphere. For both the group data (n = 5, data for each observer is normalized to the mean percept duration at intermediate contrast) and the individual observers, dominance durations are longest when observers perceive the brightest possible dots in the foreground and de dimmest possible dots in the background. A decrease of the dot luminance of one of the layers of a balanced high luminance stimulus does not decrease the durations of the episodes when this layer is perceived in the foreground. Instead, it increases the durations of the opposite perceptual interpretation. If however the dot luminance of a balanced low luminance stimulus is increased, this only influences the duration of the episodes when the varied dot luminance is perceived as the foreground (see also figure 4).

Figure 3 demonstrates that starting with a high luminance stimulus, a decrease in dot luminance of one of the two dot surfaces only affects the mean percept duration of the 3-D percept with the alternative surface (consisting of the brighter dots) in front. For example the percept of a sphere with high luminance dots in both the front and back has approximately the same mean dominance duration as the percept of a sphere with low luminance dots in the front and high luminance dots in the back, but significantly shorter average percept durations than the percept with high luminance dots in the front and low luminance dots in the back.

In other words, decreasing the stimulus strength of one perceptual interpretation does not influence the average dominance duration of this percept but it does influence average dominance durations of the opposing percept. A statistical analysis of the effect confirms that decreasing the stimulus strength of a perceptual interpretation has no significant effect on the dominance duration of the same percept (F(2,33) = 0.16, p = 0.8525) but does have a significant effect on the dominance duration of the opposite percept (F(2,33) = 17.41, p<0.0001). As in binocular rivalry, the opposite holds true for low luminance stimuli. Here an increase in stimulus strength does increase the mean dominance duration of the same percept (F(2,33) = 16.92, p<0.0001) while leaving the dominance durations of the opposite percepts unaffected (F(2,33) = 0.62, p = 0.54). This pattern of effects is present for all individual observers as well as the group data (figure 3).

**Figure 4:**
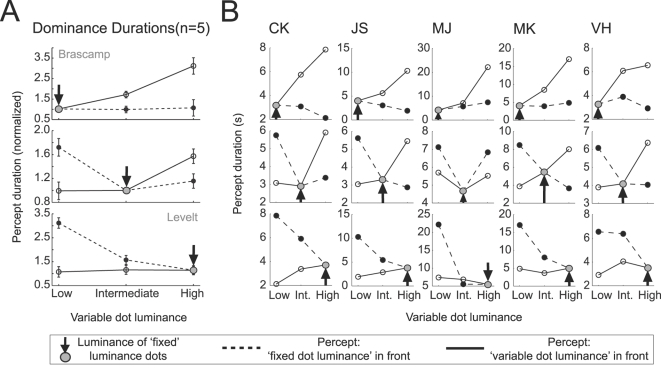
Starting with a balanced stimulus of low, intermediate or high luminance (indicated with a grey dot and an arrow) the dot luminance of one of the two layers is manipulated while that of the other remains fixed. Mean percept durations are plotted for episodes when the sphere is perceived with the fixed dot luminance surface in the foreground (dotted lines) or with the variable dot luminance in the foreground (solid lines). For both the average group data (A) and the individual observers (B) manipulations of dot luminance mainly affect the mean dominance durations of the percept with the brightest dots in the foreground. Error bars represent the standard error of the mean.

Figure 4 plots the effects of a manipulated dot luminance on mean dominance duration in a different way for the group data and individual observers. Starting with a fixed dot luminance (arrow), the dot luminance of one surface is varied (solid line) while that of the other is fixed (dashed line). Figure 4 clearly demonstrates that changes in mean dominance duration predominantly occur for the percept with the brightest dots in front. This effect is independent of which dot luminance is manipulated and consistent with our revised second proposition.

### • Proposition 3: “Manipulating the stimulus strength of one perceptual interpretation of a bistable stimulus will influence the average rivalry reversal rate.”

Levelt's third proposition directly followed from his first and second propositions. It states that increasing stimulus strength increases the predominance of the corresponding stimulus by reducing the mean dominance duration of the other stimulus rather than increasing its own mean dominance duration. This automatically results in higher reversal rates when the strength of one of the two stimuli is increased.

**Figure 5:**
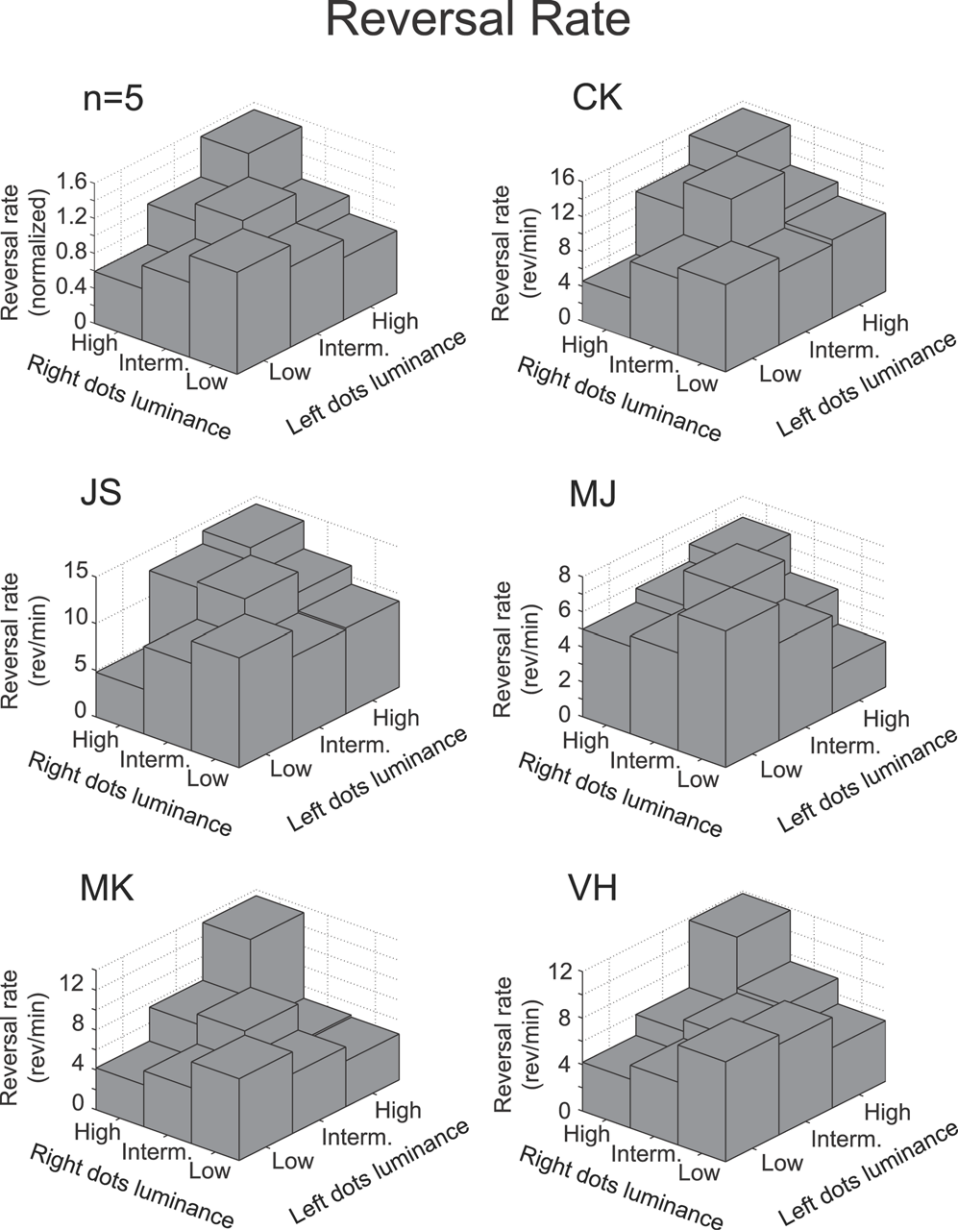
Reversal rates as a function of dot luminance of the two motion-defined surfaces. For the average group data (n = 5) the reversal rates of individual observers were normalized to the mean reversal rate over all conditions. Logically following from the demonstrated effect of dot-luminance induced percept probability on the mean dominance durations (fig. 3 & fig. 4) these plots demonstrate that for both the group data and the individual observers' reversal rates decrease when the luminance difference between the two surfaces increases. Furthermore, reversal rates for balanced stimuli increase when the dot luminance, and thus stimulus strength, increases.

Following the same line of reasoning our revised second proposition predicts that decreasing the dot luminance of one of the surfaces in a high luminance sphere would result in an increase of the dominance durations of the percept with the other (brighter) surface in front, leading to a lower reversal rate. Figure 5 demonstrates that this is indeed the case (group data is normalized to the overall mean reversal rate for an observer). The results for individual observers in figure 5 are a little noisier but the same pattern is clearly present. Statistical analysis of this effect revealed that both decreasing the dot luminance of the rightward moving dots (F(2,12) = 18.42, p<0.0001) and the leftward moving dots (F(2,12) = 24.49, p<0.0001) significantly decreased the reversal rate.

Our revised second proposition also predicts that an increase of the dot luminance of one of the surfaces in a low luminance sphere causes longer mean dominance durations of the percept with these dots in the front while hardly influencing the dominance durations of the alternative percept. Naturally this would also result in a decrease of the reversal rate. Figure 5 demonstrates that this is indeed the case and a statistical analysis demonstrated that for both dot surfaces this effect was significant (Left: F(2,12) = 11.56, p<0.001; Right: F(2,12) = 21.34, p<0.002).

Note that reversal rates can also *in*crease as a result of increasing the stimulus strength. If we start off with a stimulus consisting of one high- and one low-luminance dot-surface and we increase the dot luminance of the low-luminance surface, our second proposition predicts that the average dominance duration of the manipulated percept remains unchanged whereas that of the fixed percept decreases resulting in an increase of reversal rates. Figure 5 demonstrates that this is indeed what happens.

### • Proposition 4: “Increasing the general stimulus strength of a bistable stimulus will increase the average rivalry reversal rate.”

Until this point we have focused on changing the dot luminance in one of the two layers to manipulate the strength of the perceptual interpretation with these dots as the front surface. A test of proposition 4 however requires increasing the general strength of the stimulus. Changing the dot luminance of both surfaces with the same amount might be used to accomplish this manipulation. Figure 5 demonstrates the effect of stimulus strength on reversal rate with the bars on the diagonal of the plot. Increasing the stimulus strength does indeed increase the rivalry reversal rate (group data: F(2,12) = 4.24, p<0.05). Whereas balanced manipulations of dot luminance are unlikely to change the strength of ‘sphereness’ of the stimulus, it will influence the neural dynamics (e.g. adaptation speed) at earlier neural levels where the individual dots or surfaces are processed. The differences between subjects present in figure 5 probably reflect differences in their individual neural dynamics at these non-rivalry stages.

## Discussion

Binocular rivalry and perceptual rivalry provide unique windows on visual consciousness. Since perception alternates vividly in the absence of stimulus changes, the alternations can only result from the internal mechanism that shapes subjective experiences [25]. However, it remains unclear how similar these internal mechanisms are for binocular rivalry and perceptual rivalry. We have shown that crucial constraints for binocular rivalry models inspired by Levelt's four propositions can just as well be applied to the perceptual rivalry of a bistable rotating structure-from-motion sphere. Predominance shifts towards the strongest perceptual interpretation (I), only the mean dominance of the strongest perceptual interpretation is influenced by dot luminance-based changes in percept probability (II), reversal rates change consistent with dominance duration (III) and the reversal rates increase if dot luminance of all the dots is increased (IV). The validity of the revised second proposition in particular implies that visual competition in perceptual rivalry involves an active process of cross-inhibiting neural populations with computational principles much like we find in binocular rivalry. Thus, although the neural input to the computational mechanism of rivalry may stem from different cortical neurons and different cognitive levels the computational principles just prior to the production of visual awareness appear to be common to the two types of rivalry.

There is considerable evidence supporting the idea that perceptual outputs in binocular and perceptual rivalry are at least partially based on a common computational mechanism. Percept durations under continuous viewing conditions are distributed similarly [4] and their drift and serial correlation are also comparable [3]. Quantitatively, observers attempting to hold one percept as long as possible through voluntary control affect percept durations in perceptual rivalry more than in binocular rivalry, but the qualitative dynamic aspects are similar [5], [6], even in terms of the individual fit parameters of percept duration distributions [26]. It has also been reported that observers with slow perceptual switches in one bistability paradigm are also slow switchers in another paradigm [27], [28]. If stimuli are presented with intermittent blank periods, binocular and perceptual rivalry exhibit similar qualitative effects of stimulus timing on the percept sequences [7], [8], [9] and they are comparably influenced by voluntary control [9]. Eye movements affect the two types of rivalry in a qualitatively different way. They play a greater causal role in producing perceptual alternations in binocular rivalry than in perceptual rivalry [29]. This qualitatively different effect of eye movements and the quantitative differences with voluntary control and stimulus timing are consistent with the idea that binocular rivalry is a more low-level type of rivalry than perceptual rivalry [5], [6], [9], [29].

Our current findings, together with the studies mentioned in this paragraph, suggest that although binocular and perceptual rivalry may arise at different cortical levels, which causes quantitative differences [1], the computational rules that eventually produce perceptual output may be common (causing qualitative similarities). Note that we talk about common computational principles, not common neural machinery. Indeed, multiple bistable attributes of single binocular or perceptual rivalry stimuli undergo independent switching dynamics, suggesting that attribute-specific rivalry occurs in parallel at different levels of visual processing [30]. Our view is in line with a recently developed physiologically and mechanistically plausible model for visual rivalry [8], which is developed in terms of minimal neural activity. In this minimal model, even a single neural stage —distinguishing this model from other existing models— of rapidly inhibiting but slowly adapting percept representations can qualitatively explain all experimental findings in perceptual and binocular rivalry to date. Quantitative differences between types of rivalry can be explained in this model with different gain factors resulting from various pre-rivalry processing stages [9].

Any computational model of visual rivalry needs to be tested against experimentally established characteristics. Levelt's four propositions [14] are probably the best-known critical tests for models of binocular rivalry (recent examples are [13], [31], [32], [33], [34], [35]). It would be very useful to know if theories that have been developed to understand binocular rivalry could also be applied to other forms of rivalry. Levelt's four propositions make an excellent starting point to resolve this issue with respect to the alternation process in visual rivalry. For plaid rivalry, a manipulation of the stimulus strength of only one perceptual interpretation has already been claimed to result in behavior consistent with Levelt's second proposition [36], [37], but a detailed and systematic analysis was never reported. Our study offers the first complete and systematic test of perceptual rivalry against Levelt's four propositions revealing that all rules regarding contrast in binocular rivalry (with inclusion of the revised second proposition) can -without any fundamental modification- be applied to dot luminance in bistable structure-from-motion.

The independent manipulation of stimulus strengths mentioned in Levelt's original propositions has long hindered a systematic application of the propositions to perceptual rivalry where we have only one stimulus. It is however questionable whether this independent manipulation of stimulus strength is essential. The ongoing debate about what is rivaling during binocular rivalry primarily focuses on competition between information from the two individual eyes, the two stimulus patterns or a combination of the two [38], [39], [40]. Regardless of this debate, the competition clearly takes place between neural representations rather than between stimuli and the conflict leading to visual rivalry first presents itself when populations of neurons start coding for mutually exclusive perceptual interpretations. Without putting any claims on the exact content or location of this conflict, it seems likely that the most active neural population will ‘win’ the competition and eventually shape conscious perception. Increasing the stimulus strength of one of the two stimuli in binocular rivalry will increase the activity of the neural population coding for the corresponding percept, thereby increasing its chances to win the competition. Since this automatically decreases the probability that the opposing neural population wins the competition, it illustrates that even a unilateral manipulation of stimulus strength in binocular rivalry is still a relative manipulation at the relevant level of competing neural representations.

Our current findings are in line with previous studies that suggest that structure-from-motion is constructed through surface representations [41], [42], [43] and that the rivalry in a bistable structure-from-motion sphere takes place between the two surfaces competing for the ‘front-location’ in their depth ordering [23]. Our dot luminance manipulations bias the sphere stimulus towards the interpretation with the brightest surface in the front. The exact mechanism that establishes the bias is not crucial to our findings. Possible explanations could be that brighter objects are perceived to be closer to the observer [19] or that lower contrast dots are perceived to move slower [44]. The surface-based interpretation of structure-from-motion rivalry is consistent with the recent finding of surface based attentional modulation of neuronal activity in the area MT of the monkey brain [45] and the existence of a motion after effect specific for surface depth order [46].

In conclusion, we have shown that perceptual rivalry in bistable structure-from-motion stimuli complies with all four of the Levelt-inspired propositions, much like binocular rivalry does. Our findings do not indicate that all relevant processes underlying binocular and bistable structure-from-motion take place at the same neural level. However, the strong similarities between the two do suggest that their output is produced by –at least partially- similar computational mechanisms, justifying a generalization of computational models of visual competition over binocular and perceptual rivalry.
