# Correction: General Validity of Levelt's Propositions Reveals Common Computational Mechanisms for Visual Rivalry

**DOI:** 10.1371/journal.pone.0147454

**Published:** 2016-01-14

**Authors:** P. Christiaan Klink, Raymond van Ee, Richard J. A. van Wezel

There are errors in Fig 4 of the published article. Please view the correct Fig 4 and its legend here.

**Fig 4:**
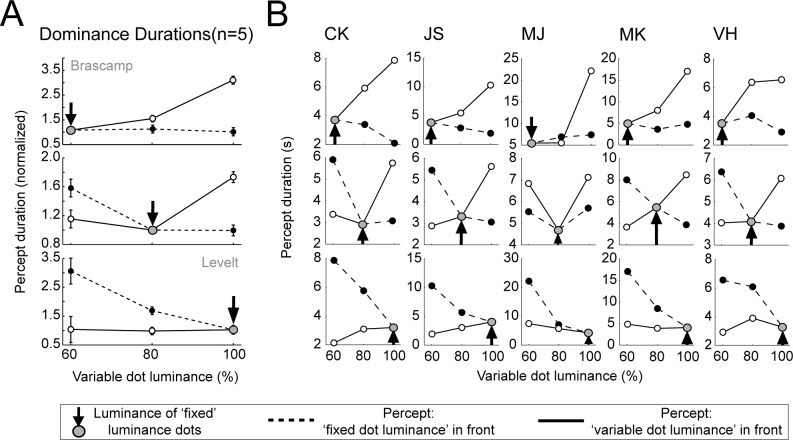
Starting with a balanced stimulus of low, intermediate or high luminance (indicated with a grey dot and an arrow) the dot luminance of one of the two layers is manipulated while that of the other remains fixed. Mean percept durations are plotted for episodes when the sphere is perceived with the fixed dot luminance surface in the foreground (dotted lines) or with the variable dot luminance in the foreground (solid lines). For both the average group data (A) and the individual observers (B) manipulations of dot luminance mainly affect the mean dominance durations of the percept with the brightest dots in the foreground. Error bars represent the standard error of the mean.
